# Dulaglutide and pregnancy: a comprehensive safety assessment using the *ex vivo* placenta perfusion and *in vitro* models

**DOI:** 10.3389/fphar.2025.1765815

**Published:** 2026-01-13

**Authors:** Sabrina Kuoni, Sara Caviglia, Alexandra Dolder, Joanna Gawinecka, Nicole Ochsenbein-Kölble, Ana Paula Simões-Wüst

**Affiliations:** 1 Department of Obstetrics, University Hospital Zurich, Zurich, Switzerland; 2 University of Zurich, Zurich, Switzerland; 3 Institute of Clinical Chemistry, University Hospital Zurich, Zurich, Switzerland

**Keywords:** BeWo cells, diabetes mellitus, dulaglutide, *ex vivo* placenta perfusion, placenta explants, placental transfer, pregnancy

## Abstract

The use of glucagon-like peptide-1 (GLP-1) receptor agonists by young populations is rapidly increasing worldwide, thereby exposing more women of childbearing age. Dulaglutide, a long-acting GLP-1 receptor agonist designed to bind to the neonatal Fc receptor (FcRn) and approved for type 2 diabetes mellitus, reflects this trend. In this study, we wanted to evaluate transplacental passage of dulaglutide and its potential effects on placental function. Transplacental transfer was investigated both with human placenta perfusions, a model for term transfer and expressing FcRn; and with permeability assays across BeWo b30 cell layers, a model for the early pregnancy placental barrier and characterised by negligible FcRn expression. Additional *in vitro* experiments were performed with human placental explants and BeWo cells. Placenta perfusions revealed minimal but consistent transplacental passage of dulaglutide (0.2%–0.7%; at 1.9 nM and 19.1 nM, over 4 hours); fluorescently labelled dulaglutide was detected within foetal villi of perfused tissue, with co-localisation to the early endosome marker Rab5. Permeability assays revealed negligible transfer. Dulaglutide exposure did not impair viability, alter the secretion of pregnancy-related hormones, nor affect glycolytic metabolism neither in human placental explants nor in BeWo cells. Our results suggest that dulaglutide has no adverse effects on placental viability and functions and is not able to cross the placental barrier at earlier gestation stages. However, its ability to cross the placental barrier at term in minimal amounts, but consistently, indicates foetal exposure during late pregnancy, when FcRn is expressed.

## 1 Introduction

Glucagon-like peptide-1 (GLP-1) receptor agonists, first introduced in 2005, represent a novel biological approach for the treatment of type 2 diabetes mellitus (T2DM). By mimicking the incretin hormone GLP-1, they enhance insulin secretion, suppress glucagon release, and slow gastric emptying. Structural modifications or fusion to large proteins protect them from rapid dipeptidyl peptidase-4 degradation, extending their half-lives to several days (Nauck et al., 2021).

Dulaglutide (Trulicity®), approved by the FDA in 2014, is a long-acting GLP-1 receptor agonist composed of two GLP-1 analogues fused to the fragment crystallisable (Fc) region of human IgG4. Through binding to the neonatal Fc receptor (FcRn), dulaglutide achieves a half-life of 4.7 days, allowing weekly dosing (Geiser et al., 2016; Scheen, 2017). Clinical studies confirmed its efficacy in glycaemic control and weight reduction in T2DM patients, with additional cardiovascular benefits (Umpierrez et al., 2016; Gerstein et al., 2019). Compared to other antidiabetic medications, it carries a low risk of hypoglycaemia, though gastrointestinal symptoms are common (EMA, 2014; FDA, 2014).

Use of dulaglutide during pregnancy is not recommended due to limited safety data. Animal studies have shown no effect on fertility, conception or foetal malformations, but reduced foetal growth and skeletal effects at high exposure, likely secondary to maternal effects (EMA, 2014). In humans, initial observational, prospective cohort studies did not indicate an increased risk of major birth defects (Dao et al., 2024). While the large size of GLP-1 receptor agonist very likely limits their ability to cross the placental barrier and reach foetal circulation, the Fc domain of dulaglutide could enable FcRn-mediated pinocytosis–the pathway by which maternal IgG crosses into the foetal circulation (Firan et al., 2001).

Hyperglycaemia in pregnancy affected an estimated 21.1 million live births globally in 2021, approximately 10% due to pregestational diabetes, including T2DM (International Diabetes Federation, 2021; Raets et al., 2023). Pregnant women with hyperglycaemia are at increased risk for preeclampsia, macrosomia or congenital anomalies, making preconception glycaemic control essential in T2DM treatment of women of childbearing age. Since many pharmacological treatment options for T2DM, such as the GLP-1 receptor agonists, are contraindicated during pregnancy, management typically involves the switch to insulin therapy, with metformin being used as a second-line option (American Diabetes Association Professional Practice Committee, 2022; Alexopoulos et al., 2019). However, the use of GLP-1 receptor agonists, including dulaglutide, is steadily increasing among young women of childbearing age (Lee et al., 2024), potentially influencing unplanned pregnancies (i.e., approximately 50% of all pregnancies (Krischer, 2025)).

This study aims to make an initial evaluation regarding the placental transfer of dulaglutide and its potential impact on placental functionality. Ethical constraints prevent the testing of medications in pregnant women, necessitating the development and implementation of various research models in the past decades. Given the inability of any single model to replicate the complexity of human pregnancy, we employed a combination of three complementary models to assess safety from multiple perspectives. First, we utilised the *ex vivo* human placenta perfusion model, considered the gold standard for the evaluation of the placental transfer, to evaluate the placental passage of dulaglutide in term human placentas. Secondly, BeWo b30 and BeWo cells isolated from human choriocarcinoma were used as a well-established *in vitro* placental model for early pregnancy (Rothbauer et al., 2017; Dusza et al., 2023) to investigate permeability through a cytotrophoblast layer and to examine the influence of dulaglutide on hormone secretion - leptin and human chorionic gonadotropin (hCG) – as well as glucose consumption and lactate production. Finally, human placental explants were employed to assess the influence of dulaglutide on tissue viability and to supplement hormone secretion data obtained from the *in vitro* experiments.

## 2 Materials and methods

### 2.1 Human *ex vivo* placenta perfusion

#### 2.1.1 Perfusion

The perfusion was performed using fresh term placentas donated by women undergoing elective caesarean sections after uncomplicated pregnancies at the Department of Obstetrics, University Hospital Zurich. The study was approved by the Ethics Committee of the Canton of Zurich (BASEC-Nr. 2023–0011, March 7, 2023) and only placentas from women who signed the informed consent were used. All participants had to sign a written informed consent before the delivery.

The *ex vivo* human placenta perfusion was conducted as previously described by Grafmüller et al. (2013) and Kuoni et al. (2024). The placental transfer of dulaglutide was assessed in an intact, marginal placental cotyledon, where the foetal artery was cannulated first (Ø 1.2 mm cannula), followed by the vein (Ø 1.5–1.8 mm cannula). The cannulated tissue was placed in a tissue holder with the maternal side facing upwards. Three maternal artery cannulas (Ø 0.8 mm) were placed into the intervillous space of the selected cotyledon, and the emerging medium was aspirated via one maternal vein.

Maternal and foetal circulation systems were prefilled with perfusion medium (PM) (formulation described in Kuoni et al. (2024)), and flow rates were set at 3 mL/min (foetal) and 12 mL/min (maternal). The foetal PM was gassed with 95% N_2_/5% CO_2_ and the maternal PM with 95% synthetic air/5% CO_2_ and the medium temperature of 37 °C was ensured by heating plates and flow heater. With an open and a closed pre-perfusion of each 20 min the tissue was washed, and its integrity was evaluated. During the main perfusion, the maternal reservoir consisted of 100 mL PM spiked with the test compound and the connectivity control creatinine (844 μM, Sigma-Aldrich). The foetal reservoir consisted of 100 mL PM without study compounds. Perfusions were conducted for 4 hours, and samples were collected at defined time points, centrifuged (10 min, 4 °C, 800 x g) and stored at −80 °C. A perfusion was considered successful if it lasted 4 h, where the connectivity control did cross the placental barrier at the expected transfer rate (Kuoni et al., 2024; Warth et al., 2019) and foetal volume loss was ≤4 mL/h.

To assess placental transfer of dulaglutide, perfusions were conducted using dulaglutide (Trulicity®, Eli Lilly) at 1.9 nM and 19.1 nM, corresponding to the maximal plasma concentration (C_max_) and 10x C_max_. In addition, three further perfusions were carried out with AlexaFluor647-labelled dulaglutide (Proteogenix) at 19.1 nM to validate the transfer data and enable microscopic localisation of dulaglutide within perfused placental tissue. For reference, one additional perfusion with no study compound was performed.

#### 2.1.2 Adherence test

Empty perfusions were performed to evaluate the adherence of dulaglutide (labelled and unlabelled) to the tubing system. This was conducted under conditions identical to a standard *ex vivo* placenta perfusion, except that only the maternal circulation was perfused in the absence of placental tissue (Kuoni et al., 2024).

#### 2.1.3 Quantification of dulaglutide and creatinine

Dulaglutide was quantified with the Dulaglutide ELISA Kit from BMA Biomedicals, according to manufacturer’s instructions (concentration range: 1.68–1676 pM). The fluorescence of the AlexaFluor-labelled dulaglutide was quantified in the perfusate supernatants using a Flexstation II (Molecular Devices) (excitation 640 nm/emission 671 nm). Creatinine was quantified by the Institute of Clinical Chemistry at the University Hospital Zurich using the Creatinine Jaffé Gen.2 method on a Cobas 8000 from Roche Diagnostics. Concentrations measured in the perfusate samples collected during the perfusion were expressed in relation (%) to the initial concentration in the maternal perfusate.

### 2.2 Tissue processing and analysis for fluorescently labelled dulaglutide

#### 2.2.1 Tissue preparation

Immediately after perfusion with dulaglutide-AlexaFluor647, the perfused cotyledon tissue was dissected into smaller fragments and fixed overnight at 4 °C in 4% formaldehyde. The samples were dehydrated in 15% and 30% sucrose solutions. Subsequently, the tissue was embedded in OCT matrix (Thermo Scientific) and stored at −80 °C until cryosectioning. Sections of 14 μm thickness were prepared using a cryostat, mounted on slides and fixed in Delaunay-fixing solution (Morphisto).

#### 2.2.2 Immunohistochemistry

Cryosections were first washed with PBS and subsequently blocked overnight at 4 °C in blocking solution (10% goat serum, 0.3% triton X-100 in PBS). After washing with PBS, sections were incubated with the primary antibody Rab5 (rabbit anti-human, 1:1000, ab218624; Abcam) or CD34 (rabbit anti-human, 1:200, ab81289; Abcam) diluted in incubation buffer (5% goat serum, 0.3% triton X-100 in PBS) overnight at 4 °C in a humidified chamber. The following day, slides were washed with PBS and incubated for 1 h at room temperature with the secondary antibodies goat anti-rabbit AlexaFluor568 (1:200, ab150077; Abcam) for Rab5 and goat anti-rabbit AlexaFluor488 (1:200, ab175471; Abcam) for CD34. DAPI (1:2000, D1306; Thermo Fisher Scientific) and phalloidin (1:2000, A12380, Thermo Fisher Scientific) were added during the secondary antibody incubation. After final washes in PBS, coverslips were mounted using ProLong™ Diamond Antifade Mountant (Thermo Fisher Scientific), and slides were stored at −20 °C until further analysis.

#### 2.2.3 Microscopy

Images were acquired using a Leica Stellaris 5 confocal laser scanning microscope (CLSM), equipped with a white light laser and a 405 nm diode laser. For each villous sample, three regions were imaged at 20x magnification (NA 0.75), and higher-resolution close-ups were obtained at 63x magnification (NA 1.4). Z-stacks were collected at 1 µm intervals for 20x images and at 0.5 µm intervals for 63x images. Maximum intensity projections of 4 µm were generated, and image processing was performed using Imaris Viewer (Oxford Instruments).

### 2.3 *In vitro* Transwell® permeability assay in BeWo b30 cells

#### 2.3.1 Cell culture

BeWo b30 choriocarcinoma cells (RRID: CVCL_LB83) were kindly provided by Buerki-Thurnherr (Empa) with permission from Dr. Alan L. Schwartz (Washington University School of Medicine). Cells were cultured in Ham’s F-12K Nut Mix medium (Thermo Fisher Scientific) supplemented with 10% heat inactivated FBS (VWR), 100 mU/L penicillin, 100 mg/L streptomycin and 20 mM L-glutamine from Thermo Fisher Scientific.

#### 2.3.2 Monolayer characterisation

BeWo b30 cells were seeded on the apical side of Corning Transwell® polycarbonate membrane inserts (24-well format, 0.33 cm^2^, 0.4 µm pore) from Sigma Aldrich at a final cell density of 30′000 cells/well. The apical and basolateral compartments were filled with 0.2 mL and 1 mL cell culture medium, respectively and medium was changed every 2–3 days. Monolayer formation was monitored under standard culture conditions (37 °C, 5% CO_2_) by placing the inserts into a cellZscope (nanoAnalytics) where the transepithelial electrical resistance (TEER) and electrical capacitance (C_Cl_) were continuously measured for each insert and were corrected for the values of cell-free inserts.

To determine optimal culture duration, TEER and C_Cl_ values were monitored daily over a six-day period, alongside paracellular permeability assays. For these initial assays, the permeability markers, FITC-dextran (40 kDa) and sodium fluorescein (NaF) were individually applied to the apical compartment at a final concentration of 5 µM in DMEM/F-12K cell culture medium without phenol red (Thermo Fisher Scientific). Permeability was assessed after 6 h by quantifying the fluorescence of FITC-dextran and NaF (excitation/emission: 490/520 nm and 460/515 nm, respectively) in both apical and basolateral compartments. Good integrity of the cell layer was defined as C_Cl_ values between 0.5 ≤ C_cl_ ≤ 5.0 μF/cm^2^ (manufacturer instructions), stable TEER values and low permeability values of FITC-dextran and NaF (Fein et al., 2023).

#### 2.3.3 Permeability assay

BeWo b30 cells were seeded onto the apical side of inserts at a cell density of 30’000 cells per insert. Culture medium was refreshed on day one and four post-seeding. On day six, a permeability assay was initiated to assess compound transport across the cell monolayer. Dulaglutide was tested separately at two concentrations (1.9 nM and 19.1 nM), as well as in its fluorescently labelled form (dulaglutide-AlexaFluor647; 19.1 nM). Control inserts were treated with FITC-dextran (40 kDa; 5 µM), which is expected to show minimal permeability, and creatinine (844 µM), which serves as a freely permeable reference compound.

Permeability experiments were conducted in DMEM/F-12K medium without phenol red for a duration of 6 h. Samples were collected from both the apical and basolateral compartments at the end of the incubation period and stored at −20 °C until further analysis. To determine the corrected apparent permeability, each experimental condition was also assessed in parallel across cell-free inserts.

#### 2.3.4 Data analysis

$$P_{app}\left(\text{cm}/s\right)=\frac{\Delta Q/\Delta t}{AC_{0}}$$ (1)

The transfer of the drug was expressed as apparent permeability (P_app_) (Equation 1), calculated by dividing transport flux $(\frac{\Delta Q}{\Delta t})$ by the insert surface area (A) and the initial apical drug concentration (C_0_).

$$P_{c}\left(\text{cm}/s\right)=\frac{1}{\left(\frac{1}{P_{t}}-\frac{1}{P_{m}}\right)}$$ (2)

The apparent permeability across the BeWo b30 cell layer (P_t_) was subsequently corrected to the apparent permeability across an empty insert (P_m_) (Equation 2).

### 2.4 FcRn gene expression in human placental tissue and BeWo b30 cells

FcRn gene expression was determined in term human placental tissue as well as in BeWo b30 cells used in permeability assays by performing real time PCR. Fresh perfused tissue was collected immediately after the completion of the perfusion with unlabelled dulaglutide and cryopreserved in liquid nitrogen. RNA was extracted from cryopreserved tissue and from BeWo b30 cells using the RNeasy Mini Kit (Qiagen), followed by complementary cDNA synthesis using the High-Capacity RNA-to-cDNA Kit (Thermo Fisher Scientific). Gene expression was quantified in dublicate using FCGRT TaqMan Gene expression assay (Hs00175415_m1) and TaqMan Fast Universal PCR Master Mix on an Applied Biosystems 7500 Fast instrument. Expression levels were normalised to GAPDH (Hs02758991_g1) and expressed as delta Ct values.

### 2.5 Human term placental explants

#### 2.5.1 Preparation of term placental explants

The cultivation of human villous placental explants was performed based on previous work (Miller et al., 2005; Gomes et al., 2019) and was adapted with the aim of obtaining a short-term explant culture, with a stable tissue viability of 4 days. Placentas were collected from term, uncomplicated pregnancies immediately after caesarean delivery; only placentas with no visible disruptions were considered. Immediately after hand over of the tissue, the decidual tissue was removed with scissors and smaller tissue villous fragments were dissected without the chorionic tissue. The fragments were rinsed in ice-cold PBS and placed in ice-cold culture medium (DMEM/F-12K supplemented with 10% FBS and 1% penicillin/streptomycin) for transportation.

For further processing, each fragment was placed in a petri dish filled with PBS, for washing and removal of visible blood vessels. Placed in a petri dish filled with ice-cold culture medium, the explants were cut into same sized pieces (∼2 mm in diameter) and were distributed into a 24-well incubation plate, where each well contained one explant from three different prepared fragments. Filled with 600 μL culture medium, the plate was kept in a humidified incubator at 5% CO_2_ air atmosphere with daily medium changes.

#### 2.5.2 Effect of dulaglutide on term placental explants

Explants from four different placentas were prepared and the test substances were introduced on day two for additional 2 days. Dulaglutide was added at three concentrations (1.9 nM, 3.8 nM and 19.1 nM). Control wells were treated with either pure culture medium or sodium-citrate (0.49 g/L), while positive control explants received ethanol (4%) (Huovinen et al., 2022) or phloretin (100 μM) (Habtemariam, 2023). All experimental conditions were tested in quadruplicates. Supernatants were collected at day four and were stored at −20 °C for subsequent hormone and glucose/lactate quantification (as described in detail below under 2.6). Following the 2-day exposure-period, explant viability was assessed using the [3-(4,5-dimethylthiazol-2-yl)-2,5-diphenyltetrazolium bromide] (MTT) assay (Sigma-Aldrich).

#### 2.5.3 MTT viability assay

The influence of dulaglutide on metabolic viability of the explants was evaluated using the MTT assay. Explants were incubated in a 0.37 g/L MTT solution prepared in culture medium for 2 h in the incubator (5% CO_2_ air atmosphere). Following the incubation, the explants were transferred to dimethyl sulfoxide (DMSO) on an orbital shaker for 15 min to dissolve the formazan crystals formed within the tissue. The absorbance of the resulting purple solutions was measured in an absorbance reader at a wavelength of 590 nm.

### 2.6 *In vitro* BeWo cell experiments

The influence of dulaglutide on secretion of pregnancy-related hormones, leptin and hCG, as well as the consumption and production of glucose and lactate, was evaluated *in vitro* using BeWo cells (RRID: CVCL_0044). BeWo cells were purchased from (CCL-98) from ATCC and were cultured in F-12K (Kaighn’s modification of Ham’s F-12) medium (Thermo Fisher) supplemented with 10% FBS and 1% penicillin/streptomycin at 37 °C in a humidified incubator with 5% CO_2_ air atmosphere.

Cells were seeded at a cell density of 3.6 x 10^4^ cells/well in 12-well plates and cultured for 4 days prior to treatment with test compounds. Afterwards, cells were incubated for additional 4 days, with the medium refreshed after 2 days. Dulaglutide was added in triplicate at three different concentrations (1.9 nM, 3.8 nM and 19.1 nM) alongside control wells exclusively treated with F-12K medium or sodium-citrate (0.49 g/L, Merck), which matches the sodium content of the dulaglutide injection formulation. Phloretin (100 μM, Adipogen) was used as positive control due to its inhibitory effect on leptin (Habtemariam, 2023).

Leptin was quantified with an ELISA described by Malek et al. (1997), Malek et al. (2001) and adapted by Kuoni et al. (2024) and hCG with the human CG beta DuoSet ELISA from Biotechne. Glucose and lactate concentrations were quantified using Glucose-Glo^TM^ and Lactate-Glo^TM^ assays (Promega).

## 3 Results

### 3.1 Placental transfer of unlabelled dulaglutide during *ex vivo* placenta perfusion

The placental transfer of dulaglutide was evaluated at two different concentrations, namely, C_max_ (1.9 nM, n = 4) and ten times higher (19.1 nM, n = 3) over 4 h of perfusion, using creatinine as connectivity control.

**FIGURE 1:**
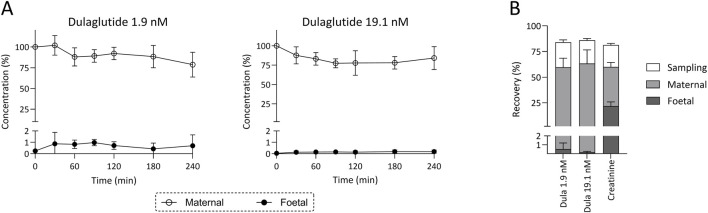
Placental perfusion and recovery of dulaglutide. Placenta perfusion profiles **(A)** obtained during human *ex vivo* placenta perfusion experiments with dulaglutide at two different concentrations (1.9 nM, n = 4; 19.1 nM n = 3). Final recovery **(B)** of dulaglutide (Dula) and the connectivity control creatinine (884 μM) across all perfusions. Data are shown as mean values ±SD.

At both concentrations, only minimal amounts of dulaglutide were detectable in the foetal circulation (0.70% ± 0.95% at lower concentration and 0.18% ± 0.14% at the higher concentration), while approximately 80% of the medication remained in the maternal compartment (Figure 1). The connectivity control creatinine confirmed effective exchange between the maternal and foetal circulations, showing consistent transfer across all experiments after 4 h of perfusion. For recovery analysis, the combined amount of dulaglutide in the maternal and foetal reservoirs, together with the quantity removed during sampling, was compared with the initially added dose. Mean recovery was 84.1% ± 11.0% at 1.9 nM and 86.0% ± 14.9% at 19.1 nM. In three empty perfusions, where dulaglutide circulated through the maternal perfusion system without connected placental tissue, its concentration decreased by roughly 15% of the initial added concentration.

As transfer of dulaglutide is supposed to be mediated by FcRn, the levels of this receptor expressed in placental tissue were determined. Placental tissue was collected from three independent perfusion experiments, and three sections from each placenta were analysed for FcRn gene expression. Quantitative PCR, using the 2^−ΔCT^ method relative to GAPDH, demonstrated expression levels of 0.056 ± 0.012, thereby confirming the presence of FcRn in the here used placental tissues.

### 3.2 *Ex vivo* placenta perfusion of fluorescent-labelled dulaglutide

**FIGURE 2:**
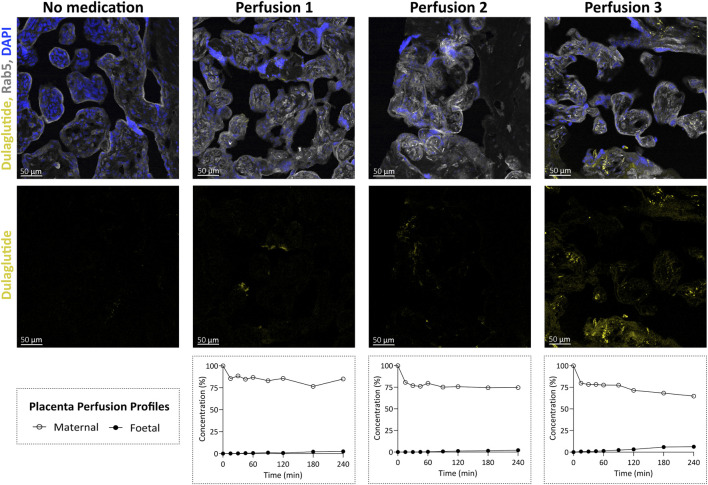
Immunofluorescence visualisation of dulaglutide-AlexaFluor647 distribution in human placental villous tissue following *ex vivo* placenta perfusion. Confocal images of perfused and unperfused placental villous tissues (foetal origin) showing dulaglutide-AlexaFluor647 (yellow), the early-endosome marker Rab5 (grey) and DAPI (nuclei, blue). The upper panels display merged channels, while the lower panels show dulaglutide-AlexaFluor647 alone. Images represent villous fragments obtained after three independent perfusions with dulaglutide-AlexaFluor647 (Perfusion 1–3) and one control perfusion without the study compound (No medication), each performed for 4 h. The corresponding perfusion profiles (shown below) illustrate relative dulaglutide concentration in the maternal and foetal perfusate over 4 h of perfusion.

In three independent human placenta perfusion experiments, the transplacental passage of dulaglutide-AlexaFluor647 was assessed by quantifying fluorescence in the foetal and maternal perfusate samples. After 4 h of perfusion, 3.6% ± 2.3% of the initially added compound was detected in the foetal compartment, while 74.8% ± 10.1% remained in the maternal circulation. Transfer rates varied across experiments, with dulaglutide-AlexaFluor647 crossing approximately three times faster in perfusion three, compared to perfusions one and two (Figure 2). The overall mean recovery was approximately 77%, which was consistent with empty perfusions, where approximately 20% of dulaglutide-AlexaFluor647 adhered to the tubing system.

In addition to the sample analysis, perfused placental tissue was immediately collected after perfusion and processed for immunofluorescence assessment to evaluate the abundance and distribution of fluorescently labelled dulaglutide within the villous tissue. For each perfusion, three different villous regions were imaged by confocal microscopy and compared to tissue from a reference perfusion where no compound was added. For image processing, brightness and contrast were adjusted equally across all images. Compared to reference tissue, all three tissues collected from dulaglutide perfusions showed detectable fluorescence within the foetal villous structures. Perfusion three exhibited the strongest and most abundant signal in the foetal villous compartment, whereas perfusion one showed the weakest (Figure 2). This was consistent with the higher dulaglutide concentrations measured in the foetal perfusate of perfusion three. In all experiments, fluorescence was most pronounced in regions adjacent to the maternal decidual tissue, initially the closest to the circulating medium.

#### 3.2.1 Co-localisation of fluorescently labelled dulaglutide and early endosomal marker Rab5 in perfused placental villi

**FIGURE 3:**
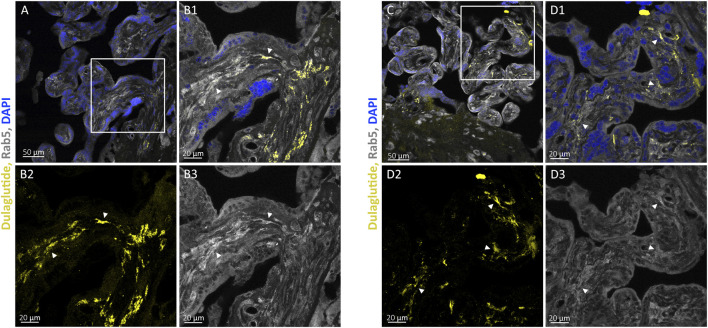
Dulaglutide-AlexaFluor647 and Rab5 co-localisation in perfused placental villous structure. Representative confocal images of placental villi obtained after two *ex vivo* placenta perfusion experiments with fluorescently labelled dulaglutide (yellow), immunostained for Rab5 (early endosome marker; grey) and counterstained with DAPI (nuclei, blue). Images were acquired at 20x magnification **(A,C)** and at higher resolution in close-up views of the marked regions at 63x magnification **(B1–B3,D1–D3)**. Panel show merged channels **(B1,D1)** as well as individual channels **(B2,B3,D2,D3)**. White arrowheads indicate sites of co-localisation.

To assess the potential uptake of dulaglutide via endocytosis, placental tissue perfused with fluorescent dulaglutide were immunostained for the early endosome marker Rab5, and co-localisation was examined using CLSM. For each tissue section, three different regions were imaged. Fluorescent signals corresponding to dulaglutide-AlexaFluor647 were more prominent within the villous stroma compared to the marginal syncytiotrophoblast layer. Overlay with Rab5 signal revealed regions of co-localisation, most notably within the central stromal compartment, indicating an association of dulaglutide with early endosomal structures (Figure 3).

#### 3.2.2 Local association of dulaglutide-AlexaFluor647 and foetal endothelial cells in perfused placental villi structure

**FIGURE 4:**
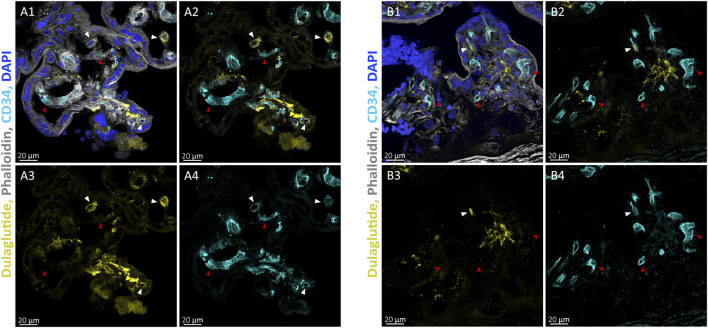
Distribution of fluorescently labelled dulaglutide relative to CD34^+^ foetal vessels in perfused placental villous structures. Representative confocal images of placental villi (foetal origin) obtained after *ex vivo* placenta perfusion experiments with dulaglutide-AlexaFluor647 (yellow). Sections were immunostained for the endothelial cell marker CD34 (cyan), and counterstain with phalloidin (F-actin, grey) and DAPI (nuclei, blue). Images from two independent tissue samples are shown at 63x magnification **(A,B)**, with individual channels displayed separately. White arrowheads indicate co-localisation sites of dulaglutide-AlexaFluor647 and the endothelial cell marker CD34, red arrowheads sites of no co-localisation.

Perfused placental tissue was further immunostained for the endothelial cell marker CD34 to investigate potential co-localisation of dulaglutide with foetal blood vessels. As demonstrated above, the predominant signal was localised within the villous stroma. Representative confocal images revealed only limited dulaglutide-associated fluorescence in proximity to foetal blood vessels (Figure 4).

### 3.3 BeWo b30 cell permeability assays with dulaglutide

Initial experiments showed that BeWo b30 cells, seeded at 30’000 cells/well, formed an intact cell layer on polycarbonate inserts, as evidenced by a marked reduction in the permeability of FITC-dextran (40 kDa) and NaF over 6 days (Supplementary Figure S1), comparable to reference values (Fein et al., 2023). Consequently, a final incubation time of 5 days was selected for subsequent assays, with C_Cl_ values of 0.86 ± 0.02 μF/cm^2^ and consistent TEER values of 21.0 ± 2.5 Ω*cm^2^.

**FIGURE 5:**
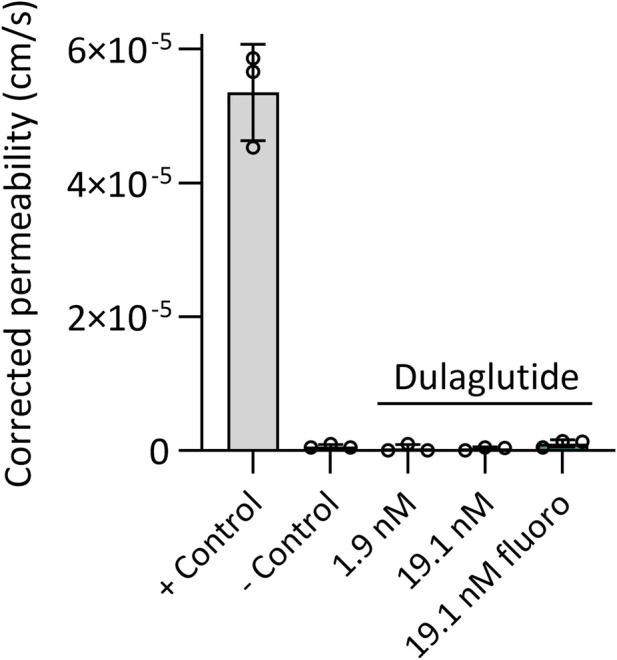
Corrected apparent permeability of dulaglutide and reference substances across BeWo b30 cell layer. The apparent permeability of dulaglutide was assessed at two concentrations (1.9 nM and 19.1 nM) as well as in its fluorescently labelled form (dulaglutide-AlexaFluor647; 19.1 nM). Creatinine (844 µM) and FITC-dextran (40 kDa, 5 µM) served as positive and negative controls, respectively. Permeability was determined after 6 h of incubation and was corrected for diffusion across cell-free inserts. Data represent mean values (n = 3) ± SD.

The transfer was evaluated across a BeWo b30 cell layer, which expresses FcRn at levels approximately 500-fold lower than measured in term placental tissue by real time PCR (Supplementary Figure S2), corresponding to a relative abundance of 1 × 10^−4^ compared to GAPDH. During three independent assays, the apparent permeability of dulaglutide (1.9 nM and 19.1 nM) and dulaglutide-AlexaFluor647 (19.1 nM) across a BeWo b30 cell layer was evaluated and corrected to the apparent permeability across cell-free inserts (n = 3). All three dulaglutide conditions showed neglectable apparent permeability of 3–10 × 10^−7^ cm/s, and were similar to the permeability of the negative control, FITC-dextran 40 kDa that was approximately 6 × 10^−7^ cm/s (Figure 5).

### 3.4 Effects of dulaglutide on the functional properties of placental explants

Different preparation steps and culture conditions were tested to establish optimal short-term culture conditions for human term explants. The highest viability was achieved when placental fragments were dissected and washed within minutes after delivery, followed by transport in cold culture medium. Using scissors instead of punchers facilitated the easy removal of bigger blood vessels. With the finalised preparation protocol, explants from three placentas showed consistent viability (99.1% ± 6.2%) after 4 days of incubation, as assessed by the MTT assay, relative to their initial viability at the start of incubation.

**FIGURE 6:**
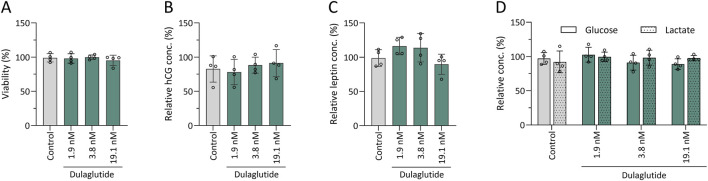
Influence of dulaglutide on human placental explant viability and functionality. Influence of dulaglutide on human placenta explant viability **(A)**, hormone secretion – hCG **(B)** and leptin **(C)** – and glucose consumption and lactate production **(D)** was investigated over 2 days. Mean concentrations (n = 4) are expressed relatively to control wells treated with culture medium ±SD and compared to control wells (0.49 g/L sodium-citrate).

Explants from four different placentas were prepared and maintained in culture. After a 2-day pre-incubation period, they were exposed to dulaglutide at three concentrations (in quadruplicate) for an additional 2 days. Viability, assessed by MTT assay, showed that dulaglutide-treated explants exhibited mitochondrial activity comparable to control explants treated with sodium-citrate (0.49 g/L) (Figure 6A). In contrast, explants treated with ethanol (4%) as a positive control showed reduced viability (reduced to 80% compared to control), confirming assay functionality. Supernatants collected at the end of incubation were analysed for leptin, hCG, glucose and lactate. Mean values indicated that dulaglutide treatment did not alter hormone secretion (Figures 6B,C) or glycolytic activity, as glucose and lactate levels remained stable compared to control wells regardless of the applied concentration (Figure 6D).

### 3.5 Effects of dulaglutide on BeWo cell functionality

**FIGURE 7:**
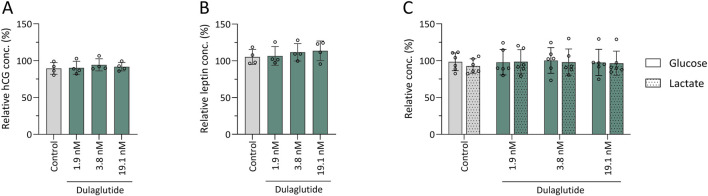
Influence of dulaglutide on BeWo cells functionality. Influence of dulaglutide on placental hormone secretion – hCG **(A)** and leptin **(B)** – and glucose consumption and lactate production **(C)** was investigated in BeWo cells. Mean concentrations (n = 4 for hormones, n = 6 for glucose/lactate) are expressed relatively to control wells treated with culture medium ±SD and were compared to control wells (0.49 g/L sodium-citrate).

BeWo cells were treated for 4 days with three different concentrations of dulaglutide (1.9 nM, 3.8 nM, and 19.1 nM) in triplicate. No changes were observed in their secretion of leptin or hCG compared with control cells treated with sodium-citrate (0.49 g/L) (Figures 7A,B). Similar results were obtained for glucose consumption and lactate production, where no change compared to control wells was observed (Figure 7C). Phloretin, a glucose uptake inhibitor with a known suppressive effect on leptin secretion, was used as a positive control. In this setting, phloretin reduced leptin secretion to approximately one-fifth of baseline levels, while glucose levels in the supernatant were nearly five-fold higher, thereby confirming assay validity.

## 4 Discussion

With the growing use of GLP-1 receptor agonists among women of childbearing age and the lack of data on dulaglutide’s safety and placental transfer during pregnancy, this study provides the first direct investigation of how dulaglutide crosses the placenta and its potential effects on placental function and viability, addressing a critical knowledge gap.

The placental transfer of dulaglutide at term was investigated using the *ex vivo* human placental perfusion model, applying two different concentrations–C_max_ and ten times higher–and validating the results additionally with a fluorescence-based approach. Across all three experimental conditions, dulaglutide, measured in the maternal and foetal perfusate, demonstrated measurable passage across the placental barrier, although transfer rates remained minimal (0.2%–3.6%). The relatively high molecular weight of dulaglutide (∼60 kDa) likely limits passive diffusion (Syme et al., 2004) suggesting that alternative transport mechanisms are required. Structurally, dulaglutide incorporates the Fc backbone of an IgG4 molecule conjugated to two GLP-1 sequences, enabling interaction with the FcRn, thereby elongating its half-life in the human body. This same FcRn-mediated interaction facilitates the placental transfer of IgG4 during late pregnancy via pinocytosis, as previously demonstrated in the *ex vivo* human placenta perfusion studies by Malek et al. (Malek et al., 1995). In this process, fluid-phase endocytosis by syncytiotrophoblasts internalises IgG4, which binds FcRn within endosomes at low pH and is subsequently released into the foetal circulation upon neutral pH (Sand et al., 2022). The observed slow transfer rate of dulaglutide is comparable to that of IgG, as shown in *ex vivo* placenta perfusion studies, where only 0.5% of IgG crossed the placental barrier after several hours (Malek et al., 1995). Similar slow transfer kinetics have also been reported for other Fc-fused proteins (Guinn et al., 2024; Porter et al., 2016).

Immunofluorescence analysis of dulaglutide-AlexaFluor647 in the perfused placental tissue confirmed the uptake of some compound into the foetal villous compartment of the term placenta, with signal intensity correlating with the low concentrations measured in the foetal perfusates. Within the villous tissue, the signal was predominantly localised in the stromal region rather than the outer syncytiotrophoblast layer, indicating passage across this cellular barrier. Co-localisation with the early endosomal marker Rab5 supports the notion that dulaglutide is internalised via an endocytic pathway. In addition, some co-localisation of dulaglutide around foetal endothelial cells was observed, suggesting translocation across the placental barrier into the foetal circulation. However, this accumulation was only observed around a minority of visible vessels, which is likely related to the slow transfer rate. The results obtained with fluorescently labelled dulaglutide are in line with those discussed above for unlabelled dulaglutide. Both were obtained in the *ex vivo* placenta perfusion model, which is considered the gold standard for evaluating drug transfer across the placenta. However, the model is limited to term placentas, thereby constraining its applicability to earlier stages of gestation, where effects on the foetal development may have stronger consequences.

FcRn expression in the human placenta, particularly in the syncytiotrophoblasts, increases progressively during the third trimester (Lozano et al., 2018; Simister, 2003). In contrast, FcRn is absent in cytotrophoblasts, the trophoblastic subtype that predominates during the early stages of pregnancy (Simister et al., 1996). The abundance of FcRn correlates with the findings that IgG transcytosis across the placenta only begins in early second trimester and increases progressively with advancing gestation, reaching its highest transfer efficacy after approximately 36 weeks (Palmeira et al., 2012). Consequently, by employing a Transwell® permeability assay using BeWo b30 cells, a well-established *in vitro* model of human cytotrophoblasts characterised by very low FcRn expression, we imitated the conditions of early gestation. Under these conditions, dulaglutide showed very low permeability, matching that of the negative control marker FITC-dextran. Taken together, the observation that dulaglutide consistently crosses the placental barrier at term, in conjunction with detectable FcRn expression in term placental tissue, while not being able to cross the cytotrophoblast layer, which shows 500x less FcRn expression, supports the hypothesis that dulaglutide transport across the placenta is FcRn-mediated, i.e., is likely to occur via transcytosis. Consequently, it is plausible that maternal treatment with dulaglutide during the later stages of pregnancy would result in higher net foetal exposure compared to administration early in gestation, when Fc-mediated transfer is expected to be absent or minimal. Minimal exposure in early pregnancy could be particularly relevant in the management of diabetes in cases of unplanned pregnancies in women taking dulaglutide, as it could mean that a comfortable time window for substitution of dulaglutide by recommended medications exist.

In addition to investigating the transplacental passage of dulaglutide, we wanted to evaluate its potential effects on placental function. The *ex vivo* placenta explant and *in vitro* BeWo cell experiments demonstrated that dulaglutide did not significantly alter the secretion of two key pregnancy-related hormones, leptin and hCG, nor did it affect glucose or lactate metabolism. These findings indicate that dulaglutide does not disrupt endocrine pathways crucial for maintaining pregnancy. BeWo cells, derived from a choriocarcinoma line, constitute a well-established trophoblast cell model. Although they do not fully replicate the complexity of primary trophoblasts, they remain widely used to investigate placental endocrine function (Dusza et al., 2023; Bode et al., 2006; Fujisawa et al., 2000). In parallel, human placental explants preserve the three-dimensional villous architecture and multicellular composition of the placenta, thereby retaining more of the *in vivo* context (Dusza et al., 2023). No single placenta model replicates the complexity of the human placenta throughout gestation. The combination of those different *in vitro* and *ex vivo* models, however, allows the balance of throughput, functional readouts and physiological relevance.

In conclusion, this study provides the first evidence of dulaglutide transfer across term human placentas and offers mechanistic insights into the placental transport of this GLP-1 receptor agonist. Across complementary placental models, dulaglutide did not impair endocrine activity or glycolytic function. However, given the demonstrated potential for foetal exposure if administered during term pregnancy, further investigations are warranted to evaluate its impact on late foetal development.

## Data Availability

The original contributions presented in the study are included in the article/Supplementary Material, further inquiries can be directed to the corresponding author.
