# Supplementary material for: Dulaglutide and pregnancy: a comprehensive safety assessment using the ex vivo placenta perfusion and in vitro models
Source: Front Pharmacol. 2026 Jan 13;16:1765815. doi: 10.3389/fphar.2025.1765815 (PMC12865208; doi:10.3389/fphar.2025.1765815)
Supplement: Supplementary file 1 [file DataSheet1.docx]

Supplementary Material

# Supplementary Figures and Tables

## Supplementary Figures


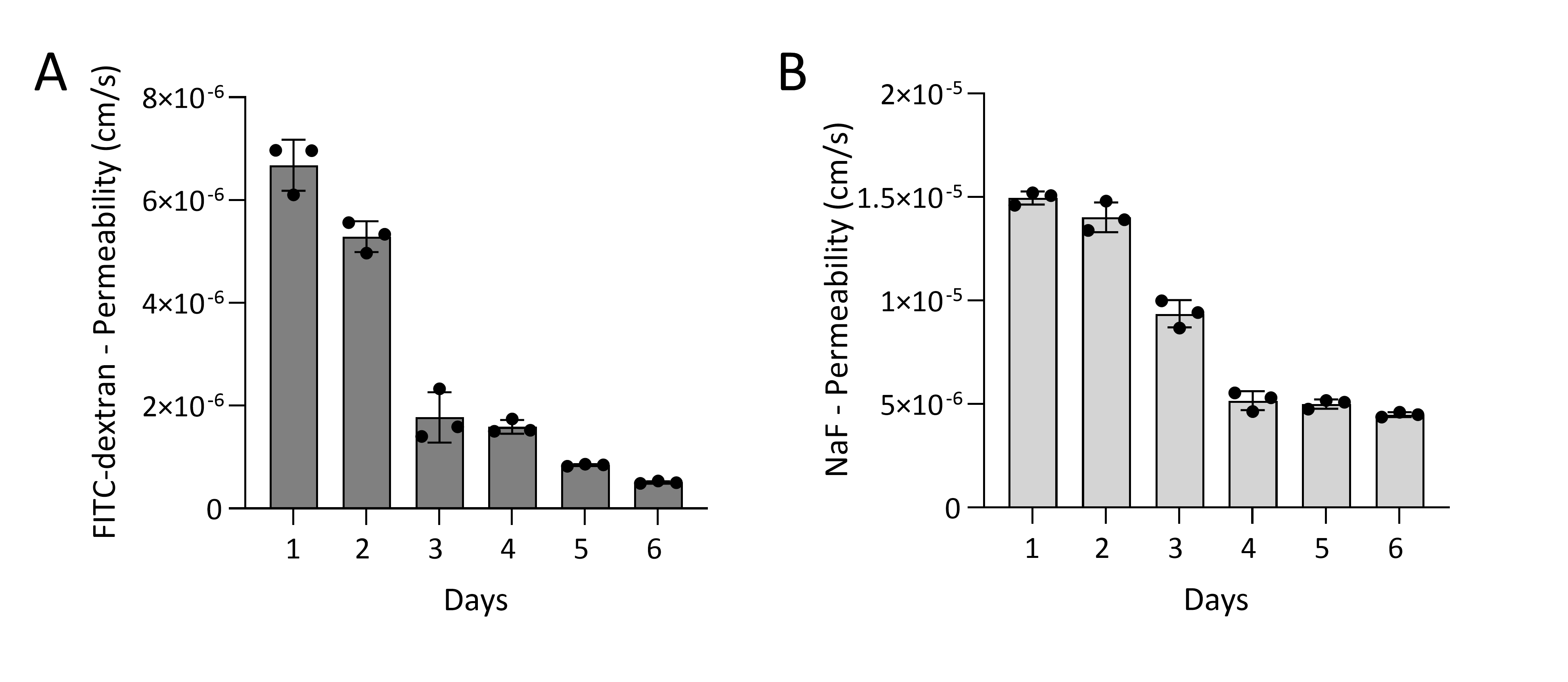


**Supplementary Figure 1: Transwell® permeability assay of reference markers.** Permeability (cm/s) of two reference markers, FITC-dextran (40 kDa) (A) and sodium fluorescein (NaF) (B), across a BeWo b30 cell layer cultured on polycarbonated Transwell® inserts. Both markers were applied apically at a concentration of 5 µM for six hours in three independent experiments. Data are presented as mean values ± SD.


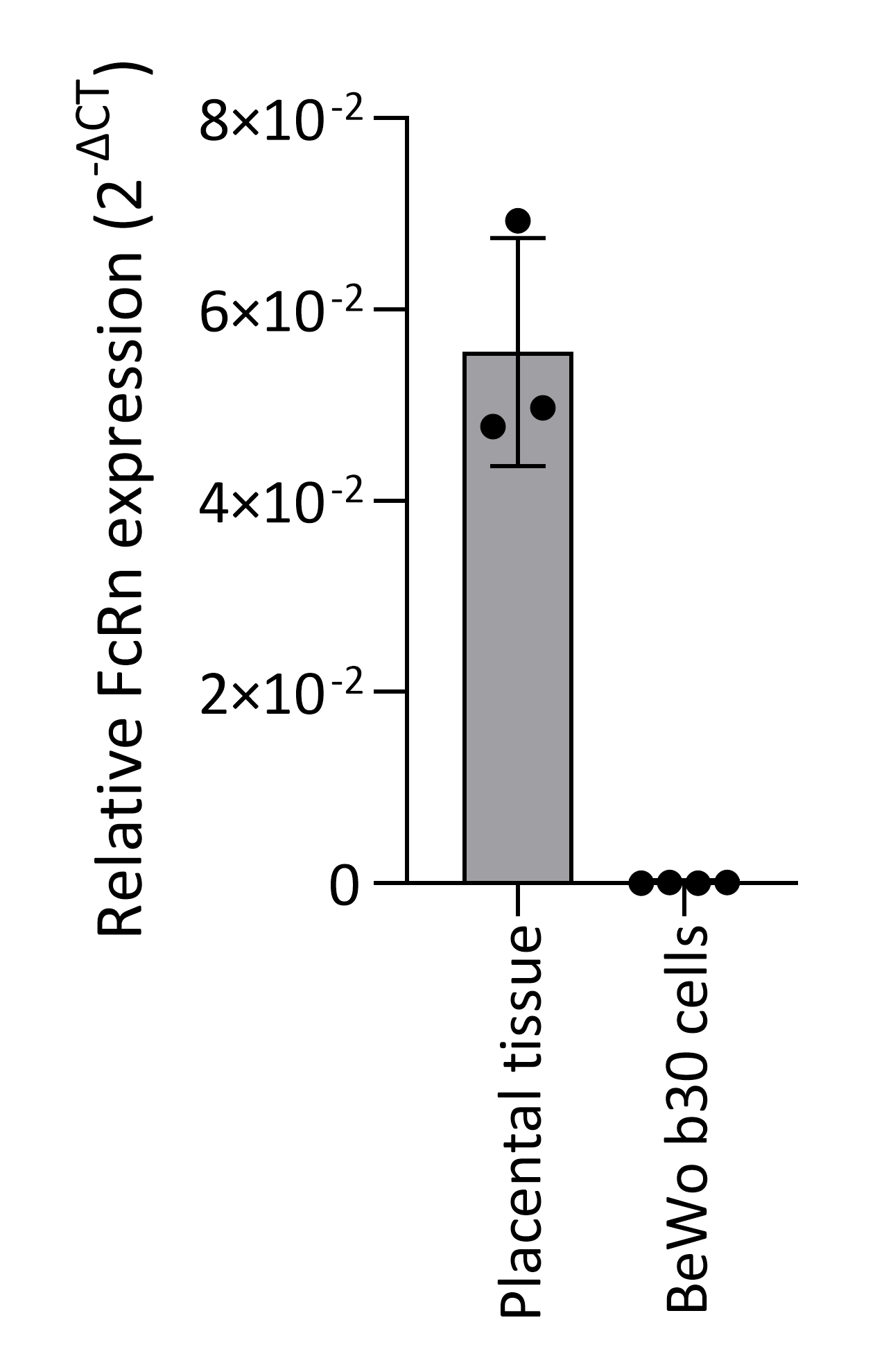


**Supplementary Figure 2: Relative expression of neonatal Fc receptor (FcRn) transcripts.** FcRn mRNA expression levels in perfused term placental tissue and BeWo b30 cells used in the Transwell® permeability assay. Gene expression was quantified with real time PCR and relative to GAPDH. Data are shown as mean values ± SD (n=3 for placental tissue; n=4 for BeWo b30 cells).
